# The Collective Leadership for Safety Culture (Co-Lead) Team Intervention to Promote Teamwork and Patient Safety

**DOI:** 10.3390/ijerph17228673

**Published:** 2020-11-22

**Authors:** Aoife De Brún, Sabrina Anjara, Una Cunningham, Zuneera Khurshid, Steve Macdonald, Róisín O’Donovan, Lisa Rogers, Eilish McAuliffe

**Affiliations:** 1 UCD Centre for Interdisciplinary Research, Education, and Innovation in Health Systems (UCD IRIS), School of Nursing, Midwifery & Health Systems, University College Dublin, Dublin 4, Ireland; sabrina.anjara@ucd.ie (S.A.); ucunningham@mater.ie (U.C.); zuneera.khurshid@ucdconnect.ie (Z.K.); steve.macdonald@ul.ie (S.M.); roisin.o-donovan@ucdconnect.ie (R.O.); lisa.rogers@ucdconnect.ie (L.R.); Eilish.McAuliffe@ucd.ie (E.M.); 2 Transformation Office, Mater Misericordiae University Hospital, Eccles St, Dublin 7, Ireland; 3 School of Medicine, University of Limerick, V94 T9PX Limerick, Ireland

**Keywords:** team training, leadership, safety culture, patient safety, teamwork

## Abstract

Traditional hierarchical leadership has been implicated in patient safety failings internationally. Given that healthcare is almost wholly delivered by multidisciplinary teams, there have been calls for a more collective and team-based approach to the sharing of leadership and responsibility for patient safety. Although encouraging a collective approach to accountability can improve the provision of high quality and safe care, there is a lack of knowledge of how to train teams to adopt collective leadership. The Collective Leadership for Safety Cultures (Co-Lead) programme is a co-designed intervention for multidisciplinary healthcare teams. It is an open-source resource that offers teams a systematic approach to the development of collective leadership behaviours to promote effective teamworking and enhance patient safety cultures. This paper provides an overview of the co-design, pilot testing, and refining of this novel intervention prior to its implementation and discusses key early findings from the evaluation. The Co-Lead intervention is grounded in the real-world experiences and identified needs and priorities of frontline healthcare staff and management and was co-designed based on the evidence for collective leadership and teamwork in healthcare. It has proven feasible to implement and effective in supporting teams to lead collectively to enhance safety culture. This intervention overview will be of value to healthcare teams and practitioners seeking to promote safety culture and effective teamworking by supporting teams to lead collectively.

## 1. Introduction

There has been increasing focus on the crucial role of leadership to promote effective patient safety. Patient safety has become a focus of research and intervention internationally as a result of major investigations in the US [1] and the UK [2] emphasising the significant impact of errors and system failures on patient safety, morbidity and mortality. Leadership is often suggested as a viable and powerful target for intervention, given its influential role in shaping healthcare cultures that provide high quality and safe care [3]. While leadership approaches have emerged that represent a shift away from the single-leader approaches (for example, transformational leadership and adaptive leadership), historically leadership development in healthcare has primarily focused on the heroic, individual-as-leader model [4], often taking individuals out of the system, training them in a university environment, and expecting that they will be equipped to deploy their newly-acquired skills upon return to the healthcare setting. It is challenging for one individual to enact change in a large complex system, and the appropriateness of this model is questionable, given healthcare delivery is almost wholly dependent on multidisciplinary team (MDT) effort [5,6].

Recent systematic reviews demonstrate that there is increasing and strong evidence for effectiveness of team-based and collective approaches to leadership in healthcare settings [7,8,9]. For instance, Wu et al. [8] found that shared leadership played a “crucial role” in fostering positive team outcomes in multiple domains, including group behavior processes, attitudinal outcomes, team cognition, and team performance (p. 60). Despite this accumulating evidence for shared and collective approaches to leadership, there is limited guidance or training on how to support teams in working more collectively to enhance safety [9]. To address this gap, we embarked on a lengthy research process to: (a) understand the challenges to working collaboratively that are encountered in healthcare teams; (b) utilise the best available evidence to co-design a targeted intervention to help teams to work collectively to improve their performance; and (c) implement and assess the impact of this intervention on healthcare teams. The Co-Lead team intervention is the result of this five-year programme of work. The intervention is currently being deployed and widely tested. Our objective in this paper is to provide a sufficiently detailed description of this open-access intervention to enable adoption and further testing in other settings in light of calls to enable the comparability and replicability of implementation and testing of interventions of this nature [9]. This paper provides an overview of the design, development, and testing of this complex intervention, describes the content and resources offered by the intervention and reflects on its contribution as well as the potential for this intervention to promote safety culture and effective teamworking through supporting teams to lead collectively.

### Teamwork and Collective Leadership

In modern healthcare settings, teamwork is integral to fostering a working environment that supports and enhances safe and high-quality person-centred cultures of care. Although not mandatory in most health systems, team training is recognised as crucial to optimise team-based approaches to the delivery of healthcare [10,11,12,13]. Team training programmes aim to equip team members with the knowledge and skills necessary to communicate, collaborate, and coordinate with healthcare professionals of various levels of experience and of diverse disciplinary backgrounds. Several healthcare team training interventions exist, TeamSTEPPS [14] (developed by the US Department of Defence and the Agency for Healthcare Research and Quality) and Crew Resource Management (adapted from the aviation industry) [15,16] being amongst the most popular. While team training has proven beneficial for individual staff members, teams and healthcare organisations [10,16], there is an absence of training packages aimed at promoting safety culture and team effectiveness through enhancing collective leadership skills among team members. This novel collective leadership focus was a deliberate and critical element of our work, because of the multidisciplinary nature of modern healthcare teams and the fact that traditional leadership models have been implicated in patient safety failures [2,17]. 

A collective leadership approach may be defined as one that recognises leadership is not necessarily the sole responsibility of one individual, but may be considered a team property, where roles and responsibilities are shared as the task demands [18]. While there is accumulating evidence for the benefit and value of this more inclusive and collective approach to team outcomes in healthcare [6,8,9], there was a dearth of guidance on how to develop collective leadership in practice. The Co-Lead intervention was designed to address this gap by creating a set of resources that teams could self-deliver to support the development of the team’s skills in collective leadership. The intervention aimed to collectively improve teams’ safety culture and performance. During development, we adhered to Salas et al.’s key principles for effective team training in healthcare [13]. Among these eight principles are: (i) identification of critical teamworking competencies; (ii) emphasising teamwork over task work; (iii) recognising one size does not fit all; (iv) providing opportunity to apply learning; (v) ensuring relevance to transfer environment; (vi) ensuring timely, relevant feedback; (vii) evaluating outcomes and learning; and (viii) reinforcing designed behaviours [13]. We will reflect further on the alignment of Co-Lead to these principles in our discussion of the intervention.

A review of the evidence on leadership research in healthcare concluded that contextual, organisational and team-based elements have a meaningful impact on leadership in practice [19]. Therefore, to account for the impact of context on collective leadership, a realist evaluation was adopted to test the intervention. Realist evaluation is a methodological approach that acknowledges the role of context when implementing complex interventions. It is a theory-driven approach that explores generative causation through exploring the psychological mechanisms that trigger specific intervention outcomes in certain contextual conditions [20]. This supported the investigation of the impact of the resources offered by the intervention on four heterogeneous teams to help understand what worked for whom, to what extent, how and in what circumstances. 

## 2. Materials and Methods 

### 2.1. Development of Co-Lead

We undertook a co-design process to inform the development of the collective leadership intervention. In the healthcare context, co-design in healthcare involves the equal partnership of healthcare professionals, those with lived experience of a healthcare system or service (such as patients, families and carers) and the other stakeholders and experts relevant to the specific problem or service (e.g., researchers, IT professionals, etc.) [21]. To inform the design of this intervention, we partnered with a team of twenty-one people that included health systems researchers, healthcare professionals, managers, and patient representatives to co-design the Co-Lead intervention. Experts in collective leadership, team training, healthcare delivery, human factors, and implementation science also contributed their expertise. We held six half-day co-design workshops with one full final day over a seven-month period. At these workshops, frontline staff, managers, patients, and experts contributed their knowledge and expertise to inform, identify, and prioritise relevant content and topics that were agreed to represent crucial components of a team training intervention to promote collective leadership for safety (in line with Salas et al.’s principle [13]). Topics included experiences of teamworking, frustrations and barriers to effective teamwork, patient safety and safety monitoring, feasibility of training modalities and types of, and the measurement of, impact and outcomes. The research team informed these sessions through extensive literature reviews on key topics including collective leadership interventions, safety culture in healthcare, and case studies of effective teams working collectively. Additionally, the research invited expert presentations on safety to contribute to the co-design team discussions. A paper describing this co-design process in detail, including steps in the process, inputs, strategies to enable collaboration during sessions, and an overview of the workshop aims, content, and co-design outcomes is available [21]. The result of the co-design process was an agreed template and set of priorities to inform, build and test the final Co-Lead intervention. We have also drawn on (with appropriate permissions) existing evidence-based interventions or team training programmes and materials.

### 2.2. The Co-Lead Intervention: Overview and Module Descriptions

**Figure 1:**
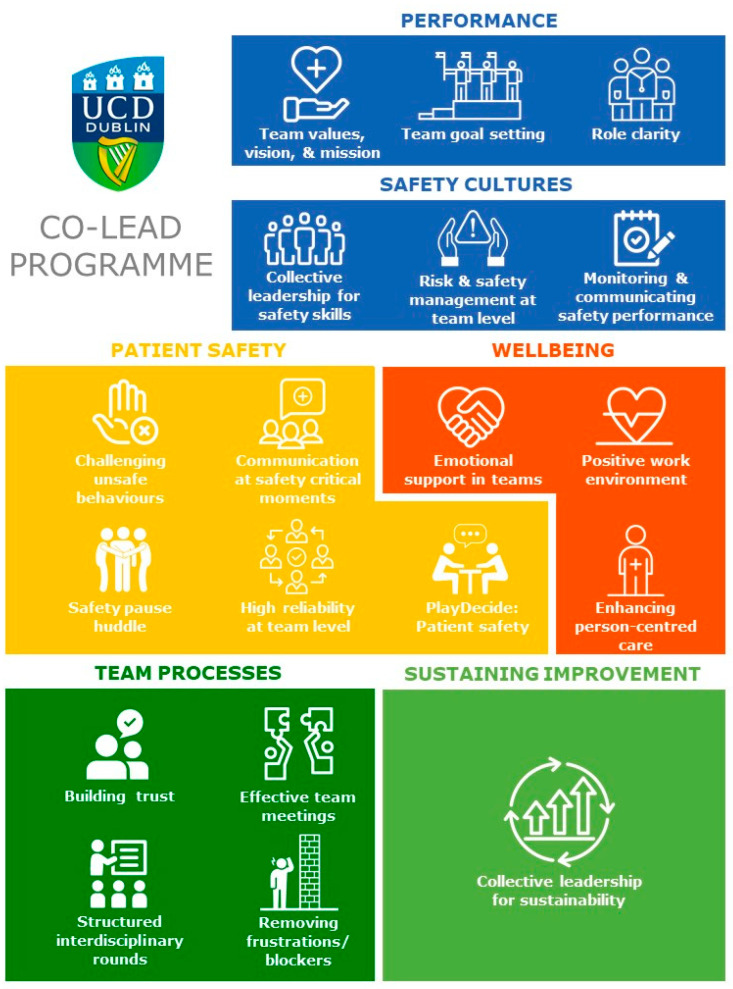
Co-Lead Programme modules.

The full intervention may be downloaded as an open access resource from www.ucd.ie/collectiveleadership. It comprises a series of modules designed to enhance collective leadership in multidisciplinary healthcare teams to promote safety cultures. These take the form of team workshops, in the team’s workplace setting, each lasting approximately one hour. There are initially six “core” modules, focusing on team performance and safety culture. These introduce the principles and outcomes of collective leadership and provide tools to achieve them. The focus of these core modules is on team performance and safety cultures. Teams may then select an additional two modules from thirteen “targeted” modules grouped into 4 main themes: team processes; well-being; patient safety; and sustaining improvements. Each module focuses on a specific area for improvement (all employing a collective leadership approach), based on what teams feel is most relevant and useful to help them achieve their goals (Figure 1). Given the Co-Lead intervention is aimed at all types of MDT, this flexibility was cited as important by the co-design team and therefore incorporated into the intervention design. This ensures the intervention does not adopt a “one size fits all approach” and can be adapted appropriately as required by the needs and priorities of teams [13]. This also allows teams to opt not to focus on practices already embedded, e.g., safety pause. The hour-long workshop structure arose since the co-design team noted that sessions longer than one hour were not practicable during a typical healthcare team’s working day. As the training is focused on the team learning and applying learning together [13], and it is neither practical nor feasible for an entire team to be away from the care delivery setting, the intervention was designed to be delivered within the workplace setting.

Each module session has a corresponding set of instructions to support session delivery. These guides enable team members to facilitate the sessions themselves, requiring no advance preparation. Open access supports available for download include materials for facilitators, a session outline, and handouts (where applicable). In some cases, there are also links to download other materials such as presentations or tools for use during the session. Adhering to Salas et al.’s principles, activities are embedded to enable team members to immediately practice learning and to support rapid transfer of learning to practice [13]. Furthermore, teams are encouraged and supported to collect meaningful measurements of improvement targets, enabling the team to reflect on performance outcomes and feedback and how to sustain new process and desirable behaviours [13].

## 3. Results

### 3.1. Intervention Mechanisms of Action

We employed a mixed methods realist evaluation in the pilot testing of the Co-Lead intervention [22]. This involved a multiple forms of data collection, including non-participant observation of team sessions, one-on-one interviews with team members and a survey to evaluate collective leadership, team working and safety culture. The realist evaluation approach required careful consideration and unpacking of the constituent elements of the intervention, the resources it offered, how it was anticipated to impact on participants and the expected outcomes, based on the extant literature. In collaboration with experts in the field of collective leadership, safety culture, quality improvement and health systems research, we developed a programme theory to understand and test how the intervention was received and the resulting impact of its implementation. This programme theory is described in detail elsewhere [18] and has been tested and refined through multiple methods of data collection [23], lending support to the mechanisms and outcomes hypothesised to occur as a result of a team’s engagement in the Co-Lead programme.

**Figure 2:**
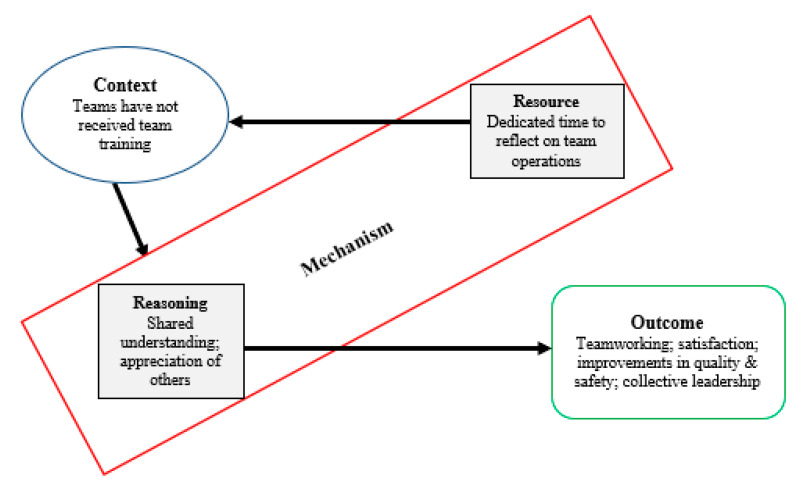
Explanatory programme theory of the how Co-Lead intervention operates.

Table A1 (see Appendix A) summarises the Co-Lead modules, provides a brief overview of the aim of each one, and consistent with the realist approach to understanding how an intervention operates [20], describes the resource mechanisms offered by the intervention and the psychological mechanisms triggered in responses to the resources offered [24]. A resource mechanism refers to the component of the intervention introduced into the context and the reasoning mechanisms relate to the participant’s implicit reactions and (unobservable psychological) reasonings in response to the intervention resources [24]. For instance, in Figure 2, the theory depicted proposes that the intervention (as a package) offers teams dedicated time to reflect on how the team operates. This is the new offering or resource provided by the Co-Lead intervention and when that resource is introduced to the context in which teams are working, it triggers the reasoning mechanisms of a shared understanding and a greater appreciation of other team members, their roles, and expertise [23]. This in turn promotes intervention outcomes including enhanced teamworking, staff satisfaction, improvements in quality and safety and the practice of collective leadership. 

We conducted a similar mapping process for each intervention module, identifying the resource mechanisms they offer (e.g., knowledge, skills, support, etc.) that may help trigger the reasoning mechanisms that lead to the desired outcomes relating to the enactment of collective leadership in teams. This process ensured the topics and priorities identified by the co-design team were linked to the final intervention design and to targeted individual and team outcomes. This resource mapping process was conducted independently by five members of the research team to ensure interrater reliability in the identification of mechanisms of resource and action. A high degree of agreement was achieved. Table A1 displays the results of this mapping process and identifies some examples of the targeted psychological mechanisms and behavioural outcomes of specific modules. 

### 3.2. Realist Evaluation of Intervention

While the focus of this paper is to outline the development and key mechanisms of action underpinning the intervention, this section reflects briefly on the findings of the realist evaluation. The realist evaluation explored the impact of the implementation of the Co-Lead intervention across four heterogeneous healthcare teams, ranging in size from small cross-organisational teams to large unit-based teams in large urban teaching hospitals. The intervention was successfully implemented in all four cases. We found that the intervention promoted a positive internal team environment, fostered the recognition that partnership is required for effective patient care and a more collective mindset was reported. This in turn led to improvements in teamworking, team performance and quality and safety [23]. This resonates with previous research identifying the importance of a positive internal team environment and the development of shared mental models and reflective practice to promote teamworking and collective leadership [8,25]. The provision of structured intervention materials to support and promote inclusivity and interdisciplinary working was strongly linked to enhanced empowerment, motivation and a shared sense of responsibility for team performance. The intervention’s collective focus aimed at highlighting expertise on the team helped to dissolve the barriers between professions on teams and enhanced psychological safety. Through creating a culture of psychological safety, participants developed interpersonal trust, felt better equipped to share leadership, were more open to voicing opinions, and senior leaders were more willing to listen and seek input [23].

## 4. Discussion

The healthcare environment requires training supports that align with contemporary demands and realities and that support the modernisation of healthcare delivery. In a review of the evidence base for leadership in healthcare by West and colleagues, they described leadership as the most influential factor in shaping organisational culture [3]. This highlights the importance of leadership as a target for quality and safety in healthcare. Despite strong evidence for its efficacy in improving staff satisfaction, team performance and organisational outcomes [10,11,13,16], healthcare has been slow to adopt team training. Given the high-risk and complex nature of healthcare delivery and the increasing complexity of patient presentations, multidisciplinary teamwork is required more than ever to help mitigate risk and adverse outcomes, and to optimise quality and safety of healthcare delivery and patient outcomes. The Co-Lead intervention has been designed with the remit of improving safety culture and team performance through the promotion of collective leadership in teams. In this discussion, we reflect on the co-design and development process and contextualise this work with reference to Salas et al.’s key principles for teamwork training development in healthcare settings [13].

Whilst there are many good examples of team training interventions to improve team performance, no existing programme focused on collective leadership as a means to promote safety and performance. We sought to harness existing resources and develop new intervention modules to meet this need. Key to the effective design of a team training intervention is ensuring its relevance: dedicating time to confirm that its design and resources meet the needs of the targeted population (i.e., frontline healthcare teams). Through bringing together a diverse group of researchers, healthcare professionals, managers, and patients, and adopting a co-design approach, we collectively identified critical teamwork competencies. Additionally, by grounding the interventions within the competing demands of healthcare teams we ensured that the realities of practice guided intervention content and structure.

Secondly, the co-design of the intervention prioritised teamwork competencies over task-based competencies, helping to ensure the broader relevance of the intervention. Aligned with this was the recognition that team training had to be flexible; beyond the six core modules, teams can tailor the intervention to suit their own prioritised needs and goals. Broad teamworking competencies, including communication, coordination, and collaboration, are emphasised through a collective leadership lens and by highlighting the need for individual and collective responsibility for the team’s quality and safety performance. In addition, where feasible, we sought to enable participants to practice newly acquired skills (e.g., in communication) through guided and practical experiences during training sessions. This approach helps to ensure that training is perceived as relevant and transferrable to the work environment [13].

The content and structure of the intervention enables team members to develop a sense of ownership for team operations, supported by shared leadership approach towards improvement. The creation of dedicated time for team reflection fostered a shared mental model among team members, created a psychologically safe environment where individuals felt secure in voicing their opinions, demonstrating that team members were more willing to offer solutions and “rise to the occasion to exhibit leadership” [26]. This resonates with previous research demonstrating that when collective leadership is practiced, team members are more likely to contribute ideas and share information [27]. Consequently, teams collaboratively and successfully implemented quality and safety initiatives (e.g., safety huddles).

The importance of a strong internal team environment to promote collective leadership has been established [8,28]. Elements underpinning this environment include a shared purpose, social support, voice and team trust [28]. Impactful efforts towards collective action are driven by foundational relationships, with aligned vision and values [29]. Collective identity is also particularly relevant to the development and enactment of collective leadership [30]. Evidence from the realist evaluation indicates that the Co-Lead intervention can foster the development of a shared mental model among team members, which can enhance coordination among team members.

Finally, the Co-Lead intervention emphasises the importance of meaningful measurement and metrics that are relevant to teams themselves. We have modules specifically to support teams to understand what is being measured by the organisation and the wider healthcare system. The intervention also helps teams to learn how they can use clinical outcome and process data to determine and improve how they operate as a team and to determine how they are performing against their key objectives over time.

In our testing of the impact of the Co-Lead intervention, a mixed-methods realist evaluation of four case studies was adopted to explore what works for whom, how, and under what circumstances. Eight context–mechanism–outcome configurations (theories) were extrapolated that explain the mechanisms triggered to drive outcomes in particular contexts [23]. This provides the first evidence for the positive impact of Co-Lead on individual (staff and patients) and team-level outcomes, including key quality and safety metrics. The next phase will explore the impact of the intervention at the organisational level and will assess the impact of team training on organisations’ safety and key performance indicators. 

## 5. Conclusions

Given the highly interdependent nature of healthcare teams, collective leadership can be considered even more important for healthcare settings [8]. In light of the strong evidence base in support of collective leadership as a means to improve team performance [8,9], it is imperative that health systems move from the traditional practice of focusing on the individual as a leader and to consider a stronger focus on the leadership potential of teams This paper presents a robust, evidence-based, co-designed intervention that addresses many of the challenges being encountered by healthcare teams in their daily efforts to deliver safe, high quality care. The co-designed, flexible nature of the intervention ensures it can be easily utilised by any team or organisation that recognises the importance of a collective, collaborative approach to improving healthcare. The pilot and formative research we have conducted with teams to date confirms the value of the intervention for team development. Although we have not completed our testing on the impact of the intervention at scale, our realist evaluation of the pilot is indicative of the intervention’s potential to positively impact team performance and patient safety. We would therefore encourage other health systems, quality improvement and patient safety researchers to collaborate with healthcare organisations to implement and evaluate this intervention in their own contexts. Just as large-scale multi-national trials are utilised to test new drugs or treatments, it is time for organisational researchers to address the many systems challenges through large-scale multi-national organisational studies. We have a collective responsibility to advance promising interventions towards the goal of delivering safe care for all.

## Appendix A

**Table A1:** Co-Lead intervention modules: summary, mechanisms of action and target behavioural outcomes.

Co-Lead Intervention Modules	Aim/Description	Resource Mechanisms Offered by Module	Reasoning Mechanisms Triggered by Module	Examples of Targeted Behaviours
**Team Values, Vision and Mission**	The aim of this session is for the team to collectively establish and agree team values, team vision, and a team mission statement. These statements will guide and drive the work and goals of the team.	Enables team time and structured process to agree and make explicit a set of values, vision, and mission statements that represent the team’s goals and ways of working.	Shared understanding Shared mental model, acknowledgement of similarities Role and process clarity Shared sense of responsibility	Collective action towards achievement of goals, e.g., monitoring, improvement efforts.
**Team Goal Setting**	The aim of this session is for the team to collectively establish the team’s goals and priorities for the coming months. These goals should align with the outputs from the previous session on creating the team’s vision and mission statements.	Opportunity to clarify and understand each other’s goals. Enabling team members to propose team goals and reach consensus on team targets, to establish a sense of ownership of team goals.	Shared understanding Shared sense of responsibility Empowerment and motivation through sense of ownership and shared responsibility for team performance.	Collaborative goal setting. Implementation of action plan for sub-teams to work towards agreed goals and improvements. Implementation of new processes or ways of working.
**Role Clarity**	This session explores the concepts of role clarity, and the way that improved role clarity can enhance the work done by teams. Participants discuss and reflect on their perceptions and expectations of the roles of different team members.	Materials enhance learning on the importance of role clarity. Provides a structure that supports staff to reflect on their role and that of others on the team.	Greater role clarity Recognising team members’ diversity in skillset, competencies, and potential to contribute Appreciation, trust, and confidence in other’s expertise	Explicitly recognising and appreciating skills of others. Collaboration across professional boundaries.
**Collective Leadership for Safety Skills**	This session focuses on collective leadership and responsibility for safety. The objective is to think about the team’s level of awareness of safety, and the safety skills that are strong in the team as well as those that need to be developed.	Materials provide evidence and learning about collective responsibility for patient care and offer the time to collectively reflect and prioritise the safety skills for improvement.	Internalisation of collective leadership concepts Understanding that all have a role to play in safety and it must be a collective effort Sense of individual and collective responsibility for team performance	Adopting new roles and responsibilities in safety management.
**Risk and Safety Management at the Team Level**	The aim of this session is to create an understanding among all team members of the nature of risk and safety at the team level and how to understand if care has been safe in the past, is safe in the present, and will be safe in the future.	Materials provide learning around risk and safety and offer the time and structure to collectively focus on current safety measures and possible risks for the team.	Shared understanding of, and responsibility for, risks and what the team can do to mitigate them. Empowerment and motivation through sense of shared responsibility for team performance.	Adopting new roles and responsibilities in safety management. Identifying risks and safety issues.
**Monitoring and Communicating Safety at Team level**	This module builds on the Risk and Safety Management module, to provide team members with a structured tool and overarching perspective on the ways in which they can track the team’s safety performance.	Time and opportunity for staff to collectively decide a wide range of performance and improvement measures specific to the team that would be meaningful for them to understand/improve performance. Materials provide a structure to drive improvement.	Shared understanding Empowerment/motivation to improve Shared sense of responsibility through encouraging ownership of safety measures to monitor.	Adopting a role in data collection or analysis of safety metrics identified as important to the team. Providing feedback to team members on safety performance.
**Effective Team Meetings**	This module facilitates teams to collectively discuss and agree on the best structure to make the most effective use of meeting times.	Materials provide team members with tools to support effective team functioning. Time and opportunity to collectively decide how to support/improve future meetings.	Shared understanding Sense of shared responsibility Sense of empowerment and control	Attendance at and contribution to team meetings. Information sharing.
**Removing Frustrations/ Blockers**	Build-up of frustrations and barriers in everyday work in healthcare can reduce team and individual efficiency and contribute to safety issues. This module helps teams identify and find possible solutions to the frustrations and barriers commonly occurring in their working practice.	Material provides structure to collectively reflect and identify problems and troubleshoot team specific operations. Provides safe space and dedicated time to discuss barriers, frustrations, blockers to team processes.	Shared mental model of way of working and challenges from various perspectives Appreciation of expertise of others Empowerment and motivation to support others due to shared burden Understanding that partnership is needed for effective patient care	Collective action towards implementation or de-implementation of processes or ways of working. Problem solving as a team.
**Building Trust**	This module provides a space where team members can get to know each other better and build trust through creation of an environment where they can share concerns and provide mutual support during times of difficulty.	Providing a safe space and dedicated time to get to know team members, share ambitions and stories, identify strengths and weaknesses, and build mechanisms of support.	Trust and confidence in others’ expertise Psychological safety Shared experience of the trust exercise, understanding the importance of communication in building trust and the impact of broken trust.	Seeking support and advice from other colleagues. Anticipating and supporting others’ needs. Learning from mistakes.
**Structured Interdisciplinary Rounds**	Structured interdisciplinary rounds (SIDRs) are a way for interdisciplinary teams to enhance their teamwork and collaboration, by strengthening communications between members around care plans for patients.	Materials provide the evidence and learning to heighten staff awareness of the benefits of inclusiveness. Provides time and opportunity for staff to collectively decide how to best implement the initiative and tailor it to their needs as a team.	Shared understanding/appreciation of the role and skills of others Individual and collective responsibility Shared understanding of how patients benefit from good interdisciplinary work, barriers that limit good interdisciplinary work, how to mitigate these barriers. Person-centred care	Implementation of SIDRs. Attendance from all relevant professionals. Speaking up and providing input to collaborate towards development of patient care plans.
**Challenging Unsafe Behaviours (CUSS) [14,31]**	Staff may occasionally be concerned when they witness unconventional or unsafe practices in the workplace but may lack the structure and tools to raise the issue with colleagues. This module provides a standardised method to support interdisciplinary communication which can be used to speak about safety concerns when they arise and encourage all staff to challenge unsafe behaviour.	Group exposure and practice of graded assertiveness method for communicating safety concerns. Agreement on a standard method for raising concerns within the team to ensure team members will be heard/understood.	Enhanced trust and psychological safety Empowerment Sense of shared responsibility Understanding that partnership needed for effective patient care Consensus and shared understanding of CUSS words (shared mental model)	Speaking up and voicing safety concerns. Seeking input from others. Using agreed CUSS terms when communicating about safety. Effective communication between team members.
**Communication at Safety Critical Moments (ISBAR_3_) [32]** #	Safety-critical moments regularly arise during clinical practice, for example during staff handovers. In this module, teams become familiarised with a structured and focused tool to enhance communication of important information during such times.	Group exposure to tools that facilitate focused communication between team members to deliver information in a structured and effective way. Understanding of the importance of clear, standardised communication	Sense of responsibility to team members and patients Understanding that partnership is needed for effective patient care	Use of ISBAR communication tool in daily practice.
**Talking about Safety (PlayDecide serious game) [33]**	In this module, team members engage in structured discussions on patient safety and error reporting, using a "serious game" learning tool that has been co-designed with input from health professionals, patients, and researchers. The game creates a safe space to encourage deep discussion about patient safety to develop a shared understanding of the importance of error reporting in strengthening patient safety culture.	Offers staff a safe structure to learn through other people’s real experiences and acknowledge challenges inherent in safety management and reporting.	Enhanced trust and psychological safety Empowerment Sense of shared responsibility	Reporting of safety events. Team reflexivity and learning from safety events and near misses (time for reflection and learning).
**Safety Pause Huddles [34]**	This module familiarises team members with the Safety Pause, which is designed to facilitate the sharing of critical information among teams to maximise patient safety following clinical handovers, with the goal of adopting it for use in everyday practice.	Offers the evidence and learning to support the Safety Pause Huddle. Provides a tool to identify and highlight safety concerns to the team. Time and a structure to collectively decide how to use the tool as a team. Demonstrates importance of regular, focused communication	Shared understanding Shared sense of responsibility Empowerment Internalisation of collective leadership concepts; shared sense of responsibility for team	Implementation of huddles into practice (e.g., during clinical handover). Improved MDT communication.
**High Reliability at the Team Level**	This module focuses on building high reliability among teams, to ensure delivery of high-quality care in a consistent manner, and with minimal errors, despite healthcare’s complex and challenging work environment.	Provides staff with evidence and learning to achieve higher collective safety awareness through reflecting individually and collectively on the reliability of the team, the factors that impact this reliability of the team and how to improve.	Understanding that partnership needed for effective patient care Shared mental models and shared understanding of how cooperation and coordination between team members can help achieve better team reliability.	Collaboration to ensure reliability in care processes. Identify opportunities for improvement. Adapting to challenges.
**Developing a positive work environment**	This module focuses on engaging the whole team to find solutions that will help foster a positive work environment which will in turn provide the support necessary to reduce stress, avoid burnout, and improve job satisfaction among staff members.	Provides affirmation of the importance of caring for self and team and a structure that enables the team time to reflect and collectively develop improvements.	Shared understanding and ownership of the initiatives and the actions to improve the work environment. Sense of shared responsibility Motivation and empowerment Job satisfaction, feeling valued	Implementation of initiatives to develop positivity and improve experience for staff and patients.
**Emotional Support in Teams**	This module focuses on the ways in which team members can create an environment of support during challenging times, and introduces a tool which can be used by line managers, colleagues, and peers to hold meaningful and supportive conversations with staff after an adverse event has occurred.	Provides practical guidance to support colleagues after a traumatic event, time to practice using the model and an opportunity to collectively reflect and agree on how to best support each other.	Enhanced trust and psychological safety Sense of feeling equipped to support team members emotionally when the need arises. Sense of shared responsibility Feeling supported	Showing support and concern for others. Role blurring and task sharing behaviours.
**Enhancing Person-Centred Care**	This module focuses on developing awareness among health professionals of the importance of featuring the patient as the focal point in the care that they provide and working collectively to build a person-centred environment in their workplace.	Emphasising humanity in care and offers teams the time to reflect on their own care, providing a structure that encourages team members to incorporate person-centred thinking into their daily practice	Shared understanding that partnership needed for effective patient care Motivation Job satisfaction and engagement Empathy for patient and staff experience	Implementation of initiatives to develop positivity and improve experience for staff and patients.
**Sustaining Improvements**	This module focuses on the next steps after teams have taken part in the Co-Lead intervention, and wish to carry forward any useful tools, information, and lessons learned.	Offers team time and structure to reflect on achievements, progress, and enables the team to collectively plan for and ensure sustainability of changes.	Shared sense of responsibility, purpose. Internalisation of collective leadership concepts Sense of achievement Team motivation Confidence in enhanced knowledge and skills in collective leadership	Putting plan or processes in place to support the team to sustain improvements. Successful maintenance of new ways of working.

***Notes***: References are included where intervention components have been adapted from or aligned to other interventions or initiatives. # The SBAR tool was developed for the US Navy and was adapted for healthcare by Leonard et al., Kaiser Permanente, CO, USA.
